# Cancer immunotherapy: potential involvement of mediators

**DOI:** 10.1080/09629359791659

**Published:** 1997-06

**Authors:** S. Ben-Efraim

**Affiliations:** 1 Department of Human Microbiology Sackler Faculty of Medicine Tel-Aviv University Tel-Aviv 69978 Israel

## Abstract

The description of a cell-free soluble anti-tumour factor by Carswell *et al*. in 1975 (*Proc Natl Acad Sci USA*, 72: 3666–3670) was followed by a long series of experimental and clinical investigations into the role of cell-free mediators in cancer immunotherapy. These investigations included research on the effects of macrophage–derived eicosanoids (cycloxygenase and lipoxygenase derivates of arachidonic acid) and of monokines such as tumour necrosis factor-α, interleukin-1 and granulocyte–monocyte–macrophage–colony stimulating factor) and of lymphocyte products: interleukins and interferons. The investigations yielded information on the effects of various factors on macrophage and T-cell activation *in vitro*, determination of direct anti-tumour properties on animal and human tumour cells *in vitro* and on therapeutic effectiveness in tumour-bearing individuals either alone or in combination with other therapeutic factors and their production by tumour cells. During recent years much effort has been dedicated towards the use of the tumour cells transfected with cytokine genes in the preparation of cancer vaccines. Cycloxygenase products (prostaglandins) were usually assumed to inhibit expression of anti-tumour activity by macrophages and an increase in their production in cancer patients was considered as a poor prognostic index. Lipoxygenase (leukotrienes) products were assumed to exhibit antitumour activity and to induce production of IL-1 by macrophages. Interleukins 2, 4, 6, 7, 12 and the interferons were extensively tested for their therapeutic effectiveness in experimental tumour models and in cancer clinical trials. The general conclusion on the use of cell-free mediators for cancer immunotherapy is that much still has to be done in order to assure effective and reproducible therapeutic effectiveness for routine use in the treatment of human neoplasia.


Mediators of Inflammation, **6**, 163–173 (1997)

### Cancer immunotherapy: potential involvement of mediators

S. Ben-Efraim

Department of Human Microbiology, Sackler Faculty of Medicine Tel-Aviv University, Tel-Aviv 69978, Israel
Fax: (+972) 3 6409160
Tel: (+972) 3 6416682

**The description of a cell-free soluble anti-tumour factor by Carswell *et al.* in 1975 (*Proc Natl Acad Sci USA*, 72: 3666–3670) was followed by a long series of experimental and clinical investigations into the role of cell-free mediators in cancer immunotherapy. These investigations included research on the effects of macrophage-derived eicosanoids (cycloxygenase and lipoxygenase derivates of arachidonic acid) and of monokines such as tumour necrosis factor-α, interleukin-1 and granulocyte–monocyte–macrophage-colony stimulating factor) and of lymphocyte products: interleukins and interferons. The investigations yielded information on the effects of various factors on macrophage and T-cell activation *in vitro*, determination of direct anti-tumour properties on animal and human tumour cells *in vitro* and on therapeutic effectiveness in tumour-bearing individuals either alone or in combination with other therapeutic factors and their production by tumour cells. During recent years much effort has been dedicated towards the use of the tumour cells transfected with cytokine genes in the preparation of cancer vaccines. Cycloxygenase products (prostaglandins) were usually assumed to inhibit expression of anti-tumour activity by macrophages and an increase in their production in cancer patients was considered as a poor prognostic index. Lipoxygenase (leukotrienes) products were assumed to exhibit anti-tumour activity and to induce production of IL-1 by macrophages. Interleukins 2, 4, 6, 7, 12 and the interferons were extensively tested for their therapeutic effectiveness in experimental tumour models and in cancer clinical trials. The general conclusion on the use of cell-free mediators for cancer immunotherapy is that much still has to be done in order to assure effective and reproducible therapeutic effectiveness for routine use in the treatment of human neoplasia.**

**Key words:** Cancer immunotherapy, Cytokines, Eicosanoids, Interferons, Interleukins, Leukotrienes, Prostaglandins

### Introduction

The field of cancer immunotherapy began approximately 100 years ago with rather 'naive' attempts to use anti-tumour antibodies raised in various animals for treatment in human sarcoma patients.[1,2] At around the same time Coley was probably the first (1891, cited in reference 3) to suggest that cell-free filtrates of bacteria might posses anti-tumour activities. Macrophages are known to produce and secrete derivates of arachidonic acid and cytokines (such as TNF-α and Interleukin-1 (IL-1). T and B cells produced and released a long series of interleukins; both macrophage and T and B-cell products are involved in the interaction between the immune system and tumour cells in a number of ways: direct anti-tumour activity, activation of immunocompetent cells *in vivo* and/or *in vitro*, and changes in their production *in vivo* in tumour-bearing hosts. Finally, it was shown that tumour cells themselves might secrete some of the above mentioned products and develop a means to resist the anti-tumour activity of various biologically active products.

© 1997 Rapid Science Publishers

The data accumulated on the activities of macrophages and T-cell products against tumour cells helped to devise certain immunotherapeutic protocols first in experimental animal tumour systems and afterwards in human neoplasia. The immunotherapeutic protocols were based on either treatment with cell-free products alone or in combination with other treatments. They also helped in devising ways to activate *in vitro* immuno-competent cells for therapeutic uses *in vivo*.

The aim of this review is to discuss the potential involvement of cell-free mediators in cancer immunotherapy. On reviewing their anti-tumour activities it should be mentioned that some of these mediators are also involved in inflammatory processes and that their production is closely interelated.

### Eicosanoids and Cancer

Cycloxygenase (prostaglandins) and lipoxygenase products have been used in experimental tumour systems and are involved in human neoplasia.

### Prostaglandins

It has generally been assumed that prostaglandins (especially $PGE_2$) inhibit anti-tumour activity of macrophages. Thus a $PGE_2$ inhibitor, indomethacin, enhanced the macrophage cytostatic activity *in vitro* against MOPC-315 murine plasmacytoma cells.[4] Indomethacin stimulation of macrophage cytostasis was inhibited by $PGE_2$[5,6] and this enhancement of anti-tumour activity was also reported *in vivo* against Ehrlich murine tumour cells.[7–10] The effect of endogenous and exogenous prostaglandins on macrophage functions was described: culture conditions that caused increased $PGE_2$ production by activated macrophages resulted in an inhibition of their tumoricidal activity whereas production of high levels of $PGE_2$ by resident and elicited macrophages was associated with an increase in their tumoricidal activity.[11] In another work,[12] it was reported that a subcutaneous injection of polyacrylamide beads in mice induced a population of immature macrophages which became fully cytostatic to syngeneic P815 plasmacytoma when stimulated *in vitro* by LPS. Blocking of PGE synthesis by indomethacin prevented the effect of LPS and addition of $PGE_2$ did not reverse the indomethacin effect but inhibited the macrophage-mediated cytostatic activity.[12] Suppression of macrophage-mediated tumour cytotoxicity was correlated with an increase in secretion of prostaglandin from the macrophages of breast cancer patients.[13,14] Elevated prostaglandin production in human breast cancer was considered a marker of high metastatic potential for neoplastic cells, the increase in PG production occurred early in the course of breast cancer and decreased later in the course of tumour development.[15] It seems that $PGE_2$ production by human monocytes is by a subset of cells other than the cells which produce IL-1.[16] It should also be mentioned that cancer cells also produce $PGE_2$ and in this context it was reported that the amount of $PGE_2$ released by cancer cells which metastasized into the liver of tumour-bearing rats was higher than that of cells metastasizing into the kidney.[17] An increase in prostaglandin levels in cancer patients was also reported:[18] of plasma prostaglandin F levels in cases of tumours of the female genital tract and of plasma 6-oxo-prostaglandin $F_{1\alpha}$ in cases of gynecological tumours.[19,20]

On the assumption that prostaglandins can suppress the development and expression of effector cells, clinical studies were initiated to determine the effect of piroxicam (a prostaglandin antagonist) in patients with recurrent unresectable squamous cell carcinoma or lymphoepithelial carcinoma of the head and neck.[21] Although some improvement in immune reactivity was noted, more studies to correlate the improvement in immune reactivity with therapeutic effectiveness of either prostaglandin antagonists alone or in combination with other treatments are needed.[21–23] A paradoxical effect of indomethacin on lymphokine-activated killer cell (LAK) activity in cancer patients was described: indomethacin enhanced LAK activity in patients with no distant metastases but depressed LAK activity in patients with such metastases.[24] Apparently, these effects of indomethacin are not related to the PGE inhibiting property of this compound.[24] Increased synthesis of prostaglandin by macrophages from breast cancer patients was assumed to inhibit macrophage mediated cytotoxicity.[25]

### Leukotrienes

Leukotrienes (lipoxygenase pathway of arachidonic acid) are usually considered to enhance the potential of the immune response.[26,27] Thus, leukotriene and indomethacin enhance additively the macrophage anti-tumour cytostatic function.[4,28] Leukotriene B4 (LTB4) was reported to augment human monocyte cytotoxic activity and enhance monocyte production of hydrogen peroxide, IL-1 and TNF.[26] 5-Lipoxygenase activation was also described to facilitate an IL-1 transduction signal.[29] Augmentation by leukotrienes of IL-1 production by human monocytes was also described in other reports.[30,31] Lipoxygenase specifically inhibited indomethacin stimulation of anti-tumour macrophage cytostasis[32] and reversed macrophage cytostasis, induced by the calcium ionophore A23187, towards P815 tumour cells *in vitro*.[31] Leukotriene C4 was reported to be an essential 5-lipoxygenase intermediate in A23187-induced macrophage cytostatic activity against P815 tumour cells.[33] Products of the lipoxygenase pathway were involved in human natural killer cell cytoxicity.[34]

### Cytokines

Immunocompetent cells reported to produce and secrete cytokines involved in the reaction of the organism to tumour cells were macrophages, T and B cells.

### Macrophage-derived cytokines

Biologically active products described as being involved in the interaction with tumour cells were tumour necrosis factor-α (TNF-α) interleukin-1 (IL-1) and granulocyte–macrophage colony stimulating-factor (GM-CSF).

*TNF-α*

The first description of TNF-α as a macrophage product was made by Carswell *et al.*[35] Since then, several reviews have appeared which have been concerned with the characterization and properties of this cytokine.[3,36–38] The mechanism and activity spectrum of TNF-α has been the topic of several investigations: it was reported that exposure of human cervical carcinoma cells to Concanavlin A (ConA) increased the total number of binding sites for rTNF-α but blocked the transduction of the signal for the cytotoxic response.[39] MethA sarcoma cells were found to be sensitive *in vitro* to human tumour recombinant TNF-α and expressed low numbers of TNF-α receptors.[40] TNF-α (unlike INF-γ or IL-1α), induced regression of subcutaneous MethA implants.[40] It was assumed that the primary lesion induced by TNF-α is vascular and the mechanism(s) involved in generation of specific cell-mediated anti-tumour immunity induced by the TNF-α treatment was not clear.[41] It was claimed that there are multiple pathways leading to resistance to TNF-α induced tumour cell cytotoxicity, among them production of transforming growth factors by tumour cells and amplified expression of certain oncogenes.[41] Apparently, viable activated monocytes produce other lytic factors in addition to TNF-α and IL-1 because TNF-α and IL-1 associated with plasma membranes of activated human monocytes lyse only monokine-sensitive tumour cells whereas viable activated monocytes lyse both monokine-sensitive and monokine-resistant tumour cells.[42] The killing of tumour cells by TNF-α was assumed to involve internalization of this ligand in the target cells.[43] The anti-tumour effect of TNF-α and of Interferon-γ against MmB16 murine melanoma was potentiated by macrophage colony-stimulating factor.[44] The anti-tumour activity of recombinant human TNF-SAM1 was enhanced by connecting the TNF compound to thymosin β4.[45] TNFα acted synergistically with IL-1 in inhibiting the growth of A375 cells.[46] An experimental study carried out in nude mice showed that intratumoral injection of an adenoviral vector containing radiation inducible DNA sequence of the *Egr-1* promoter linked to a cDNA encoding TNF-α (Ad.Egr-TNF) enhanced the tumoricidal action of ionizing radiation in a human epidermoid carcinoma xenograft.[47] By another experimental approach it was shown that murine tumour cells transduced with the gene for TNF-α, regressed unlike non-transduced tumour cells after an initial phase of tumour growth.[48]

The results *in vitro* and *in vivo* from experimental tumour models which indicated the anti-tumour activity of TNF-α, prompted initiation of clinical trials devised to determine the therapeutic effectiveness of TNF-α in human neoplasia. Intravenous injection of TNF-α in 18 cancer patients led to some clinical improvement in three lymphoma patients.[49] Recombinant TNF-α was given in combination with TNF-γ in phase I trials in 36 patients with solid tumours. Side-effects such as fatigue, fever and chills occurred: in one patient with melanoma there was a mixed response and in one patient with mesothelioma there was transient clearance of ascites from malignant cells.[50] Disappointing results were reported in a phase II study of recombinant human TNF-α in 127 cancer patients. The conclusion of the authors was that: “rhuTNFα does not appear to have significant anti-tumour activity”.[51] A similar conclusion was made in a phase II trial of rTNF-α in 22 patients with adenocarcinoma of the pancreas: “No objective responses were observed”.[52] A phase II study of recombinant TNF-α was also performed in a group of 26 renal cell carcinoma patients. The conclusion was: “rTNF given as described, has only modest anti-tumour activity in renal carcinoma and produces considerable toxicity. We plan no further studies of rTNF in this disease”.[53] In a phase I study including 16

evaluable patients with various types of metastatic cancer, there was evidence for anti-tumour effect in two patients.[54] The property of TNF-α as an immunomodulator was reported:[55] pretreatment of monocytes with IFN-α, IFN-γ, IL-1 or TNF-α resulted in enhanced human monocyte toxicity.

*Interleukin-1*

The production, characterization and properties of IL-1 have been summarized in several reviews.[56,57] It was stated that IL-1 is a key mediator of host response to microbial invasion and that it acts as a true hormone produced during infection and inflammation.[57] Human recombinant IL-1 (hrIL-1) induced proliferative responses of T cells in the presence of sub-optimal concentrations of mitogen and doubled the response to higher concentrations.[57] Human recombinant IL-1 induced release of IL-2 by T cells and acted as a potent inflammatory agent by inducing dermal fibroblast $PGE_2$ production *in vitro* and of fever in rabbits and mice.[57] The overall conclusion from these data was that IL-1 possesses both immunological and inflammatory properties.[57] Human Interleukin-1 acted as a cytocidal factor for several tumour cell lines[58] and promoted human monocyte-mediated tumour cytotoxicity.[59] Human monocytes stimulated with pneumococcal cell surface components produced IL-1 but not TNF, thus showing independence of production of the two cytokines.[60] As mentioned already[46] IL-1 acted synergistically with TNF-α against tumour cells. IL-1α enhanced carboplatinum anti-tumour activity against human ovarian cells *in vitro* and *in vivo*.[61] In an *in vitro* study with human melanoma cell lines it was shown that tumour cells which secrete IL-1 exhibited increased adhesion to endothelial cells.[62]

*Granulocyte–macrophage colony stimulating factor (GM-CSF)*

The effect of intravenous and intraperitoneal administration of GM-CSF was examined in a phase I trial of 13 cancer patients refractory to standard chemotherapy.[63] Administration of MG-CSF was well tolerated but no data were provided on clinical improvement.[63] In another study, it was reported that administration of GM-CSF in 24 patients with solid tumours enhanced monocyte cytotoxicity against a human colon carcinoma line but no data are given on the effect on clinical course of the disease.[64]

The partial and somehow disappointing results obtained in clinical trials with TNF-α may be due to several factors:

- TNF-α can be administered only in small amounts because higher amounts are toxic and induce severe side effects.
- TNF-α is a relatively small molecule and as such, is rapidly cleared after injection.
- It is possible that some human cells are resistant to TNF-α or develop mechanism(s) of defence against the anti-tumour activity of TNF-α.
- Anti-tumour activity *in vivo* requires more than one anti-tumour factor.
- TNF-α *in vivo* does not occur sufficient concentration in its contact with tumour cells.

In view of the results obtained in clinical trials with TNF-α, experiments were undertaken to use activated human macrophages or activated human peripheral blood for therapy. It was assumed that macrophages can act indiscriminately against immunogenic or non-immunogenic tumours, and that they might release monokines continuously *in vivo* (in addition to TNF-α and/or IL-1) which would be active against the tumour cells. It should be mentioned in this context that activated macrophages might release anti-tumour cytostatic products unrelated to IL-1, TNF-α and INF-α/β.[65] Results of *in vitro* experiments showed that peritoneal human macrophages obtained from renal patients on continuous ambulatory peritoneal dialysis (CAPD) can be activated *in vitro* by LPS to express anti-tumour activity and as such they acted *in vivo* against a human tumour implanted subcutaneously in nude mice.[66] Similarly, human peripheral blood cells from cancer patients, activated *in vitro* by IFN-γ and LPS, reacted against a human tumour growing in nude mice.[67] These results prompted clinical trials with autologous human peripheral blood monocytes activated *in vitro* and reinjected in to the cancer patients.[68,69] The therapeutic effectiveness of this procedure was limited[68,69] and probably more clinical trials are required in order to improve conditions for therapy with activated macrophages.

### Lymphocyte-derived Interleukins

*Interleukin-2*

Interleukin-2 (IL-2) is one of the main products of T cells and was extensively studied in the context of its anti-tumour activity. IL-2 was reported to increase human natural killer (NK) cell activity *in vitro* and this activity was partially reduced by monocytes due to $PGE_2$ production.[70] Murine lymphocytes cultured in the presence of IL-2 lysed syngeneic murine tumour cells.[71] The lytic activity was attributed to the occurrence of lymphocyte activated killer (LAK) cells Thy-1 + Lyt$^{-}$1-2+.[71] The described effects of IL-2 in enhancing anti-tumour activity promoted a series of clinical trials devised to determine the therapeutic effects of IL-2 in cancer patients. It was found that of 106 patients with metastatic cancer receiving LAK cells plus IL-2, eight had complete responses, 15 had partial responses and ten had minor responses.[72] The same group of researchers reported that autologous tumour infiltrating lymphocytes (TIL), cultured in the presence of IL-2, lysed melanoma tumour cells.[73] Melanoma-specific cytolytic tumour lymphocytes derived from TIL grown in the presence of IL-2, and injected together with IL-2, induced tumour rejection *in vivo* possibly by reacting against the gp100 epitope.[74] A phase I trial in a total of 31 evaluable patients with metastatic cancer of the breast, gastric cancer, colorectal cancer, melanoma, non-small cell lung cancer, osteosarcoma or renal cancer, received a combined treatment of IL-2, followed by TNF-α and indomethacin:[75] two partial responses were seen (in breast and renal cancer).[75] In another clinical trial, 16 patients with advanced renal cell cancer (stage IV) received a combined treatment of cyclophosphamide, INF-α and IL-2.[76] Two patients had a partial response, two had a minor response and three patients achieved stable disease.[76] The effect of IL-2 with or without LAK cells was assayed in another group of 71 patients with advanced renal cell carcinoma.[77] A low level of anti-tumour response was detected and the addition of LAK cells did not improve the response.[77] IL-2 administration was found to prolong survival of some metastatic renal cell carcinoma patients with no or moderate HLA-II expression and/or no or moderate macrophage presence in the primary tumour, but was not effective in patients with both high HLA-II and high macrophage expression.[78] Some potential uses of IL-2 treatment have been described: IL-2 and IL-7 augmented the cytolytic activity and the anti-tumour killing spectrum of αCD3-induced activated killer cells, and such cells were suggested for immunotherapy of non-immunogenic tumours.[79] Cytotoxicity of induced LAK cells against human leukaemia was augmented in the presence of either IFN-α, IFN-γ or TNF-α in cultures, and as such they might be more effective in treating human leukaemia.[80] Adoptive therapy of established pulmonary metastases with LAK cells and recombinant IL-2 has been reported.[81] Adoptive therapy with highly enriched NK cells was assumed to have a potential use in leukaemia.[82] It should be also mentioned that LAK cells are apparently not a unique cell type but a function of various types of cells.[83] Finally, one of the problems of IL-2 therapy is the toxicity of the compound: in this context, it was suggested that the oxygen free-radical scavenger, dimethythiourea, ameloriates pulmonary permeability and vascular leak syndrome associated with multiple-dose IL-2 therapy without inhibiting IL-2 induced anti-tumour cytotoxicity.[84] On the other hand, the tumour-associated antigen 90K and the soluble IL-2 receptor were associated with poor prognosis in human ovarian cancer.[85] Another way of promoting the anti-tumour activity of IL-2 was by gene transfer into tumour cells.[86–89] The use of a human renal carcinoma line transfected with IL-2 and/or INF-α gene has been suggested for the preparation of live cancer vaccines.[90] Combined treatment with granulocyte colony stimulating factor and IL-2 increased the survival time of nude mice bearing human ovarian cancer cells.[91]

*Interleukin-4*

Interleukin-4 was first described as B-cell stimulatory factor. IL-4 was reported to inhibit tumour growth of syngeneic mammary adenocarcinoma, plasmacytoma,[92] and renal carcinoma[93] by using cytokine gene transfer into the murine tumours. Repeated injections of small amounts of IL-4 around the tumour draining nodes of mice resulted in growth inhibition of poorly immunogenic and non-immunogenic tumours (cited in reference 94). Transfection of IL-4 into tumour cells induced release of this cytokine and it was effective, *in vivo*, against a wide range of tumour cells implanted in nude mice.[92] Similarly, treatment of established murine renal cancer by tumour cells engineered to secrete IL-4, induced specific T-cell dependent systemic immunity against the non-transfected tumour.[93] To my knowledge, IL-4 therapy has not yet been tested in human neoplasia.

*Interleukin-6 (INF-$\beta_2$)*

Interleukin-6 is induced in T cells by antigen stimulation. Purified human recombinant (rIL-6) mediated substantial reduction in the number of tumours.[95] Recombinant human IL-6 produced in *Escherichia coli* inhibited the growth of human breast carcinoma and leukaemia/lymphoma cell lines *in vitro*.[96] Anti IL-6 receptor antibody prevented muscle atrophy in Colon-26 adenocarcinoma bearing mice, thus suggesting that this antibody could be a potential agent against muscle atrophia in cancer cachexia.[97] IL-

6 gene transfer into murine syngeneic tumours inhibited growth of lung carcinoma[98] and of sarcoma.[99] IL-6 gene transfected into Lewis Lung Carcinoma tumour cells suppressed the malignant phenotype and was effective against parental metastatic cells.[98] Mice rejecting murine fibrosarcoma cells, transduced with retroviral vectors containing the murine IL-6 gene and secreting IL-6, exhibited a later resistance to challenge with wild tumour cells.[98] Aquisition of the ability to synthesize endogenous IL-6 markedly accelerated the growth of weakly tumorigenic rat urothelial cells, but did not induce a tumorigenic phenotype in non-tumorigenic cells.[100]

*Interleukin-7 (B/T maturation factor)*
Murine plasmacytoma cells transfected with the IL-7 gene produced IL-7 *in vivo* and were completely rejected in syngeneic mice by a T-cell dependent process.[101]

*Interleukin-12*
IL-12 has received considerable attention during recent years as a strong anti-tumour agent: IL-12 was reported to react *in vivo* against B16F10 tumour-bearing mice and its anti-tumour effect was inhibited by anti-IFN-$\gamma$ antibody;[102] IL-12-transfected fibroblast cells admixed with the murine melanoma, BL-6, showed that local IL-12 expression suppressed tumour growth and promoted development of specific anti-tumour immunity;[103] IL-12 engineered dendritic cells were effective *in vivo* against murine tumour cells.[104] In another report, it was shown that anti IFN-$\gamma$ antibody blocked IL-12-mediated tumour regression in mice.[105] In view of the results obtained from IL-12 therapy against murine tumours, clinical trials were initiated to determine the effect of IL-12 in human cancer. Phase I clinical trials of IL-12 gene therapy were done by direct injection of tumours with genetically engineered autologous fibroblasts.[106,107] Out of 13 cancer patients (six breast, five melanoma, and two head and neck), significant reduction in tumour size was observed in three patients with melanoma and one with head and neck cancer.[108]

*Interferon-$\alpha_{2b}$*
A clinical trial was done in renal cancer and melanoma patients by treatment with INF-$\alpha_{2b}$. Of 12 melanoma patients, four patients showed a partial response whereas eight patients progressed.[109] In the case of 35 patients with advanced renal cancer, an increase in immune response potential was observed.[109] The conclusion was that more studies are required to determine the therapeutic effectiveness of INF-$\alpha_{2b}$.[109] A human renal carcinoma line transfected with the IL-2 and/or the IFN $\alpha$ gene was suggested for use in preparation of live cancer vaccines.[90]

*Interferon-$\gamma$ (IFN-$\gamma$)*
IFN-$\gamma$ was first described as an antiviral agent and was among the first cytokines assayed for therapeutic effectiveness in human neoplasia. In one of the first clinical trials done in Hodgkin's disease patients it was reported that treatment with IFN-$\gamma$ led to an extension of the disease-free survival time.[110] Complete or partial remission in multiple myeloma by IFN-$\gamma$ treatment was also reported.[111] Remissions induced by IFN-$\gamma$ were also reported in patients with lymphocytic lymphoma,[112] in cases of metastatic breast cancer,[113] non-Hodgkin's lymphoma[112] and multiple myeloma.[113] However, in another study in a group of non-small cell lung cancer patients, INF-$\gamma$ treatment did not induce tumour regression.[114] A phase I trial on the effect of IFN-$\gamma$ treatment was conducted in six patients with lung cancer;[115] systemic side effects such as transient fever, nausea, headaches and flu-like symptoms were noted[115] but no data on therapeutic effect were given.[115] In a recent *in vitro* study it was reported that human carcinoma cell lines cultured with IFN-$\gamma$ expressed more CD80 and CD86 costimulatory molecules and that this increase in expression was inhibited by IL-10.[116] Transfer of the IFN-$\gamma$ gene into murine neuroblastoma,[117] fibrosarcoma,[118] adenocarcinoma,[119] colon carcinoma[119] and lung carcinoma[120] cell lines induced tumour inhibition in syngeneic mice. Human peripheral blood monocytes collected from cancer patients were activated *in vitro* to express anti-tumour activity in the presence of IFN-$\gamma$ and LPS.[67,68]

### Anti-tumour effects vs other functions of cytokines

Some facts which should be considered in the context of the anti-tumour activity of cytokines are discussed below.

*Relationship with inflammatory functions*
Macrophage-derived cytokines such as TNF-$\alpha$ and IL-1 are also major inflammatory mediators.[5,27,28,37,56,57] Human peritoneal macrophages from CAPD patients collected during infectious peritonitis are 'primed' to produce and secrete more TNF-$\alpha$ and IL-1 when cultured in the presence of LPS.[121,122] These data indicate that inflammation and cancer are interrelated events.

*Interactions in cytokine production*
Production and release of various eicosanoids and cytokines are closely interrelated.[37] For, example: production of IL-1, TNF-α and IL-6 by human mononuclear cells was induced by stimulatory agents such as LPS,[36,56] LPS-induced TNF-α production is inhibited by $PGE_2$,[123] endotoxin, TNF-α and IL-1 induce IL-6 production *in vivo*;[124] production of IL1 and TNF-α was induced in human blood mononuclear cells by LPS, whereas IL-6 suppressed the induction of IL-1β and TNF-α by LPS or PHA.[125]

*Production of eicosanoids and cytokines by tumour cells*
Production of eicosanoids and cytokines by tumour cells may have an influence on the effect of these products on tumour development: tumour cells might produce $PGE_2$;[126] the response of murine tumours to indomethacin therapy was directly related to their ability to produce prostaglandin;[127] tumours from cachectic mice produced both TNF-α and IL-1α *in vivo*;[128] a myeloma human cell line produced both TNF-α and IL-6;[129] leukaemic cells from patients with acute myeloid leukaemia produced both IL-6 and IL-1.[130] These are a few examples; the relationship between the ability of tumour cells to produce eicosanoids or cytokines and development of cancer is not yet clear.

*Production of eicosanoids and cytokines in the tumour-bearing host*
During tumour growth in rats cycloxygenase or thromboxane synthase was inhibited whereas C5 and C12-lipoxygenases of the alveolar macrophages were activated.[131] Macrophages derived from tumour-bearing animals suppressed activation of T cells, of NK cells, of LAK cells and of generation of tumoricidal activity in normal syngeneic splenic macrophages in cultures stimulated by LPS.[132] The secretion of IL-1, TNF-α but not of IL-6 was impaired in alveolar macrophages collected from tumour-bearing mice.[133] A decrease in production of IL-1 was also reported in peritoneal macrophages collected from sarcoma-bearing mice.[134] Production of IL-1 and TNF-α by tumour associated mononuclear monocytes from cancer patients was examined and showed that production IL-1 was suppressed whereas production of TNF-α was not affected.[135] LPS induced TNF-α production was impaired in macrophages from breast cancer patients,[136] but increased in patients with malignant brain tumours.[137] An example of correlation between production of TNF-α and $PGE_2$ by peripheral blood monocytes was seen in patients with bladder cancer: these patients had either higher TNF-α production or higher $PGE_2$ production.[138]

### Cell-free mediators as cancer therapeutic agents

### In vitro activation of cells for immunotherapy

Activation of autologous human peripheral blood monocytes by culture in the presence of LPS and IFN-γ was assayed with the aim of inducing anti-tumour cytotoxic activity in the treated monocytes and to use such activated cells for immunotherapy.[68,69] Clinical trials reported limited success.[68,69] Induction of LAK cells, and later of activated tumour infiltrating lymphocytes (TIL) by culturing in the presence of IL-2 was also suggested in a series of reports.[71–74] Clinical trials were carried out in groups of cancer patients; the results obtained indicated some therapeutic benefit in melanoma and renal carcinoma with approximately 10% clinical improvement in patients treated with both IL-2 and LAK cells.[72]

### Direct administration of cytokines in cancer patients

As previously mentioned the results of treatment with TNF-α were disappointing; injection of IL-2 with or without LAK cells was problematic due to the toxicity of the agent; the effect of IL-2 in patients with squamous cell carcinoma of the head and neck was limited to temporary regression of the tumours.[139] Another approach suggested was to counteract the activity of $PGE_2$ by using a PGE inhibitor: while some improvement in immunological functions was observed no data are yet available on the therapeutic effectiveness of this treatment.[21]

### Transfection of tumour cells with cytokine genes

This approach has been extensively investigated during recent years, firstly in experimental tumour models and later in clinical trials. The transfected tumours released the appropriate cytokine *in vitro* and in some cases induced T-cell mediated specific anti-tumour immunity. Partial success was observed in terms of tumour regression and clinical improvement.[140–142] Productive transfer of the IL-2 gene was shown for melanoma, renal cell carcinoma, neuroblastoma and acute leukaemia cell lines.[86,108,139–141] Several reviews have been published on the role of cytokines in cancer therapy: in a review published in 1989 it was concluded "The clinical results obtained with cytokines (INF-α, IL-2, TNF-α, GM-CSF, G-CSF) expected to show direct tumoristatic or tumoricidal effects have been disappointing".[143] A more optimistic view was expressed in another review: "local presence of cytokines, either injected repeatedly at the tumour site or released by cytokine-engineered tumour cells, arouses immunogenicity in apparently nonimmunogenic spontaneous tumours" and as such they might indicate "potential use of cytokines as a component of new tumour vaccines".[144] In another review, it was concluded: "Clinical applications [of cytokines] are progressing, but many trials must follow to assess precisely the multitude of potential uses of these molecules".[94] Finally, in a recent review it was concluded: "Although several years will probably be required to establish the true impact of the gene-transfer modalities … technological advances have opened prospects for the management of cancer patients".[142]

### Concluding Remarks

The 'golden' era on the role of cytokines in the fight against cancer started with the description by Carswell in 1975 of tumour necrosis factor.[35] Since then, various aspects of the interaction between eicosanoids, macrophage and lymphocyte cytokines and tumour state have been described and investigated. The various topics of investigation included:

- direct effects *in vitro* and *in vivo* of eicosanoids and cytokines on tumour cells in experimental and clinical trials;
- activation of monocytes, macrophages and lymphocytes *in vitro* by cytokines for use in autologous cancer patients;
- effect of tumour state in experimental systems and human neoplasia on the production and release of eicosanoids and cytokines;
- therapeutic effectiveness of combined treatment with activated immunocompetent cells and cytokines;
- role of secretion of eicosanoids and cytokines by tumour cells in the interaction between the tumour cells and the tumour-bearing host;
- therapeutic effectiveness of tumour cells transfected with cytokine genes. It is assumed that transfected tumour cells are more immunogenic and as such they induce a T cell mediated response against the wild type non-transfected tumour. They might also secrete cytokines *in vivo* which react against the tumour cells.

Marked progress has been made during recent years on the interaction between macrophage and lymphocyte products and tumour cells, and on their possible role in immunotherapy against cancer. However, many more clinical trials are required before these agents can be used in routine therapy of human neoplasia.

### References

1. Hericourt J, Richet Ch. De la Serotherapie dans le traitement du cancer. *C R Hebd Acad Sci* (Paris) 1895; **120**: 567–569.
2. Hericourt J, Richet Ch. Traitement d'un cas de sarcome par la serotherapie. *C R Acad Sci* (Paris) 1985; **120**: 948–950.
3. Ruff MR, Gifford GE. Tumour Necrosis Factor In: Pick E ed. *Lymphokines*. New York: Academic Press, Inc., 1981; **2**: 235–272.
4. Ophir R, Ben-Efraim S, Bonta IL. Leukotriene D4 and indomethacin enhance additively the macrophage cytostatic activity towards MOPC-315 tumor cells. *Int J Tiss Reac* 1987; **IX**: 189–194.
5. Bonta IL, Ben-Efraim S. Interactions between inflammatory mediators in expression of antitumor cytostatic activity of macrophages. *Immunology Lett* 1990; **25**: 295–302.
6. Elliott GR, Tak C, Bonta IL. Prostaglandin $E_2$ enhances, and leukotriene $C_4$ inhibits, interleukin-1 inhibition of WEHI-3B cell growth. *Cancer Immunol Immunother* 1989; **28**: 74–76.
7. Lala PK, Parhar RS, Singh P. Indomethacin therapy abrogates the prostaglandin-mediated suppression of natural killer activity in tumor-bearing mice and prevents tumor metastasis. *Cell Immunol* 1986; **99**: 108–118.
8. Lala PK, Parhar RS. Cure of B16F10 melanoma lung metastasis in mice by chronic indomethacin therapy combined with repeated rounds of interleukin-2: characterization of killer cells generated *in situ*. *Cancer Res* 1988; **48**: 1072–1079.
9. Lala PK, Parhar RS, Singh P, Lala PK. Cure of murine Ehrlich ascites tumors with chronic oral indomethacin therapy combined with intraperitoneal administration of LAK cells and IL-2. *Cancer Letters* 1990; **51**: 27–35.
10. Lala PK, Elkashab M, Kerbel RS, Parhar RS. Cure of human melanoma lung metastases in nude mice with chronic indomethacin therapy combined with multiple rounds of IL-2; characterization of killer cells generated *in situ*. *Intern Immunol* 1990; **2**: 1149–1158.
11. Snider ME, Fertel RH, Zwilling BS. Prostaglandin regulation of macrophage function: endogenous and exogenous prostaglandins. *Cell Immunol* 1982; **74**: 234–242.
12. Drapier JC, Petit JF. Development of antitumor activity in LPS-stimulated mouse granuloma macrophages. Regulation by eicosanoids. *Inflammation* 1986; **10**: 195–204.
13. Cameron DJ, Rittenbury M, Majeski J. Ability of cancer patient's macrophages to kill autologous tumor targets. Effects of prostaglandin inhibitors on cytotoxicity. *Cancer* 1984; **53**: 2053–2057.
14. Cameron DJ, Stromberg BV. The ability of macrophages from head and neck cancer patients to kill tumor cells. *Cancer* **54**: 2403–2408.
15. Rolland PH, Martin PM, Jacquemier J, Rolland AM, Toga M. Prostaglandin in human breast cancer: evidence suggesting that an elevated prostaglandin production is a marker of high metastatic potential for neoplastic cells. *JNCI* 1980; **64**: 1061–1070.
16. Khamsari N, Chou YK, Fudenberg HH. Human monocytes heterogeneity: interleukin-1 and prostaglandin $E_2$ production by separate subsets. *Eur J Immunol* 1985; **15**: 48–51.
17. Nakazawa I, Ohuchi K, Watanabe M, Tsurufuji SA. Difference in prostaglandin $E_2$ synthesis between cancer cells metastasizing into liver and kidney. *Prostaglandins Leukotrienes Med* 1985; **17**: 265–266.
18. Sanders RR, Lee WH, Koh L, Brennecke A, Jones WR. Plasma prostaglandin F levels and malignant tumors of the female genital tract. *Br J Obstet Gynaecol* 1980; **87**: 139–142.
19. Alam M, Jogee M, Macgregor WG, Dowdell TW, Elder MGF, Myatt L. Peripheral plasma immunoreactive 6-oxo-prostaglandin $F_{1\alpha}$ and gynecological tumors. *Br J Cancer* 1982; **45**: 384–389.
20. Braun DP, Harris JE, Rubenstein M. Relationship of arachidonic acid metabolism to indomethacin sensitive immunoregulatory function and PGE sensitivity in peripheral blood mononuclear cells of disseminated solid tumor cancer patients. *J Immunopharmacol* 1984; **6**: 227–236.
21. Braun DP, Taylor SG IV, Harris JE. Modulation of immunity in cancer patients by prostaglandin antagonists. In: Mitchell MS ed. *Immunity*

to *Cancer II. Progress in Clinical and Biological Research*. New York: Alan R. Liss Inc., 1989; **288**: 439–448.
22. Schultz RM, Pavlidis NA, Stylos WA, Chirigos MA. Regulation of macrophage tumoricidal function: a role for prostaglandins of the E series. *Science* 1978; **202**: 320–321.
23. Taffet SM, Eurell TE, Russell SW. Regulation of macrophage-mediated tumor cell killing by prostaglandins: comparison of the effects of $PGE_2$ and $PGI_2$. *Prostaglandins* 1982; **24**: 763–773.
24. Chao TY, Ting CS, Yeh MY, Chang JY, Wang CC, Chu TM. Effects of indomethacin on lymphokine-activated killer cell activities in cancer patients. *Tumour Biol* 1995; **16**: 230–242.
25. Cameron DJ, O'Brien P. Relationship of the suppression of macrophage mediated tumor cytotoxicity in conjunction with secretion of prostaglandin from the macrophages of breast cancer patients. *Int J Immunopharmacol* 1982; **4**: 445–450.
26. Gagnon L, Filion LG, Dubois C, Rola-Pleszczynski M. Leukotrienes and macrophage activation: augmented cytotoxic activity and enhanced interleukin-1, tumor necrosis factor and hydrogen peroxide production. *Agents Actions* 1989; **26**: 141–147.
27. Bonta IL, Ben-Efraim-S, Mozes T, Fieren MJWA. Tumour necrosis factor in inflammation: relation to other mediators and to macrophage antitumour defence. *Pharmac Res* 1991; **24**: 115–130.
28. Bonta IL, Ben-Efraim S: Involvement of inflammatory mediators in macrophage antitumor activity. *J Leuk Biol* 1993; **54**: 613–626.
29. Mugridge KG, Perreti M, Parente L. 5-lipoxygenase activation may facilitate an interleukin-1 production signal. In: Samuelson B ed. *Advances in Prostaglandins, Thrombocytes and Leukotriene Research*. New York: Raven Press, 1990; **21**: 517–520.
30. Rola-Pleszczynski M, Lemaire I. Leukotrienes augment interleukin-1 production by human monocytes. *J Immunol* 1995; **135**: 3958–3961.
31. Rola-Pleszczynski M, Stankova J. Cytokine regulation by $PGE_2$, $LTB_4$ and PAF. *Mediators of Inflammation* 1992; **1**: 5–8.
32. Hilten van JA, Elliott GR, Bonta IL. Specific lipoxygenase inhibition reverses macrophage cytostasis towards P815 tumor cells *in vitro* induced by the calcium ionophre A23187. *Prostagl Leuk Ess Fatty Acids* 1988; **34**: 187–192.
33. Hilten van JA, Ben-Efraim S, Zijlstra FJ, Bonta IL. Leukotriene $C_4$ is an essential 5-lipoxygenase intermediate in A23187-induced macrophage antitumor cytostatic activity against P815 cells. *Prostagl Leuk Ess Fatty Acids* 1990; **39**: 283–290.
34. Rossi P, Lindgren JA, Kullman C, Jondal M. Products of the lipoxygenase pathway in human natural killer cell cytotoxicity. *Cell Immunol* 1985; **93**: 1–8.
35. Carswell EA, Old LJ, Kasse R, Green S, Fiore N, Williamson B: An endotoxin-induced serum factor that causes necrosis of tumors. *Proc Natl Acad Sci USA* 1975; **72**: 3666–3670.
36. Aggarwal BB, Kohr WJ, Hass PE, *et al.* Human tumor necrosis factor. Production, purification and characterization. *J Biol Chem* 1985; **260**: 2345–2354.
37. Ben-Efraim S. Interactions between macrophage cytokines and eicosanoids in expression of antitumour activity. A review. *Mediators of Inflammation* 1992; **1**: 295–308.
38. Bemelmans MHA, van Tits LJH, Buurman WA. Tumour Necrosis Factor: function release and clearance. *Crit Rev Immunol* 1996; **16**: 1–11.
39. Aggarwal BB, Traquina PR, Essalu TE. Modulation of receptors and cytotoxic response of Tumour Necrosis Factor-α by various lectins. *J Biol Chem* 1986; **261**: 13652–13656.
40. Palladino MA Jr, Shalaby MR, Kramer SM *et al.* Characterization of the antitumor activities of human tumor necrosis factor-α and the comparison with other cytokines: induction of tumor-specific immunity. *J Immunol* 1987; **138**: 4023–4032.
41. Shepard HM, Lewis GD. Resistance of tumor cells to tumor necrosis factor. *J Clin Immunol* 1988; **8**: 333-341.
42. Ichinose Y, Bakouche O, Tsao JY, Fidler IJ. Tumour necrosis factor and IL-1 associated with plasma membranes of activated human monocytes lyse monokine-sensitive but not monokine-resistant tumor cells whereas viable activated monocytes lyse both. *J Immunol* 1988; **141**: 512–518.
43. Smith MR, Munger WE, Kung HF, Takacs L, Durum SK. Direct evidence for an intracellular role for tumor necrosis factor-α Microinjection of tumor necrosis factor kills target cells. *J Immunol* 1990; **144**: 162–169.
44. Lasek W, Wankowicz A, Kuc K *et al.* Potentiation of anti-tumor effects of tumor necrosis factor α by macrophage-colony-stimulating factor in a MmB16 melanoma model in mice. *Cancer Immunol Immunother* 1995; **40**: 315–321.
45. Noguchi K, Inagawa H, Tsuii Y, Morikawa A, Mizuno DI, Soma G. Antitumor activity of a novel chimera tumor necrosis factor (TNF-STH) constructed by connecting rTNF-S with thymosin-β4 against murine syngeneic tumors. *J Immunother* 1991; **10**: 105–111.
46. Ruggiero V, Baglioni C. Synergistic anti-proliferative activity of interleukin-1 and tumor necrosis factor. *J Immunol* 1987; **138**: 661–663.
47. Mauceri HJ, Hanna NN, Wayne JD, Hallahan DE, Hellam S, Weichselbaum RR. Tumour necrosis Factor α (TNF-α) gene therapy targeted by ionizing radiation selectively damages tumor vasculature. *Cancer Res* 1996; **56**: 4311–4314.
48. Asher AL, Mule JJ, Kasid A, *et al.* Murine tumor cells transduced with the gene for tumor necrosis-α. Evidence for paracrine immune effects of tumor necrosis factor against tumors. *J Immunol* 1991; **146**: 3227–3234.
49. Selby P, Hobbs S, Viner C, *et al.* Tumour necrosis factor in man: clinical and biological observations. *Br J Cancer* 1987; **56**: 803–808.
50. Smith II JW, Urba WJ, Clark JW, *et al.* Phase I evaluation of recombinant Tumour Necrosis Factor given in combination with recombinant Interferon-gamma. *J Immunother* 1991; **10**: 353–362.
51. Hersh EM, Metch BS, Muggia FM, *et al.* Phase II studies of recombinant human Tumor Necrosis Factor alpha in patients with malignant disease: a summary of the southwest oncology group experience. *J Immunother* 1991; **10**: 426–431.
52. Brown TD, Goodman P, Fleming T, *et al.* A phase II trial of recombinant Tumor Necrosis Factor in patients with adenocarcinoma of the pancreas: a southwest oncology group study. *J Immunother* 1991; **10**: 376–378.
53. Skillings J, Wierbicki R, Eisenhauer E, *et al.* A phase II study of recombinant Tumor Necrosis Factor in renal cell carcinoma: a study of the National Cancer Institute of Canada Clinical Trials Group. *J Immunother* 1992; **11**: 67–70.
54. Blick M, Sherwin SA, Rosenblum M, Gutterman J. Phase I study of recombinant Tumor Necrosis Factor in cancer patients. *Cancer Res* 1987; **47**: 2986–2989.
55. Philip R, Epstein LB. Tumour necrosis factor as immunomodulator and mediator of monocyte cytotoxicity induced by itself, γ-interferon and interleukin-1. *Nature* 1986; **323**: 86–88.
56. Dinarello CA. The biology of interleukin-1 and comparison to tumour necrosis factor. *Immunol Lett* 1987; **16**: 227–231.
57. Dinarello CA, Cannon JG, Mier JW, *et al.* Multiple biological activities of human recombinant interleukin-1. *J Clin Invest* 1986; **77**: 1734–1739.
58. Onozaki K, Matsushima K, Kleinerman ES, Saito T, Oppenheim JJ. Role of interleukin 1 in promoting human monocyte-mediated tumor cytotoxicity. *J Immunol* 1985; **135**: 314–329.
59. Onozaki K, Matsushima K, Aggarwal BB, Oppenheim JJ. Human interleukin-1 is a cytocidal factor for several tumor cell lines. *J Immunol* 1985; **135**: 3962–3968.
60. Riesenfeld-Orn I, Wolpe S, Garcia-Bustos JF, Hoffmann MK, Toumanen E. Production of interleukin-1 but not tumor necrosis factor by human monocytes stimulated with pneumococcal cell surface components. *Infect Immun* 1989; **57**: 1890–1893.
61. Wang Z, Lee KB, Reed E, Sinha BK. Sensitization by Interleukin-1α of carboplatinum anti-tumor activity against human ovarian (NIH:OV-CAR-3) carcinoma cells *in vitro* and *in vivo*. *Int J Cancer* 1996; **67**: 585–587.
62. Burrows FJ, Haskard DO, Hart IR, *et al.* Influence of tumor-derived Interleukin 1 on melanoma-endothelial cell interactions *in vitro*. *Cancer Res* 1991; **51**: 4468–4775.
63. Toner GC, Gabrilove JL, Gordon M, *et al.* Phase I trial of intravenous and intraperitoneal administration of granulocyte–macrophage colony-stimulating factor. *J Immunother* 1994; **15**: 59–66.
64. Chachoua A, Oratz R, Hoogmoed R, *et al.* Monocyte activation following systemic administration of granulocyte–macrophage colony-stimulating factor. *J Immunother* 1994; **15**: 217–224.
65. Lepoivre M, Boubdid H, Lemaire G, Petit JP. Cytostatic product(s) released by activated macrophages, unrelated to Interleukin-1, Tumor Necrosis factor α, and Interferon-α/β. *Cell Immunol* 1988; **115**: 273–287.
66. Ben-Efraim S, Tak C, Romjin JC, Fieren MJWA, Bonta IL. Therapy with human macrophages against a human tumor implanted in nude mice. *Med Oncol Tumor Pharmacother*, 1994; **11**: 7–12.
67. Bartholenys J, Lombard Y, Dumont S, *et al.* Immunotherapy of cancer: experimental approach with activated macrophages proliferating in culture. *Cancer Detect Prev* 1988; **12**: 413–420.
68. Andreesen R, Scheibenbogen C, Brugger W, *et al.* Large scale production of human cytotoxic macrophages grown from blood monocytes of cancer patients. *Cancer Detect Prev* 1991; **15**: 407–412.
69. Hennemann B, Scheibenbogen C, Schumichen, Andreesen R. Intrahepatic adoptive immunotherapy with autologous tumorcytotoxic macrophages in patients with cancer. *J immunother* 1995; **18**: 19–27.
70. Suzuki H, Yamashita N, Sugiyama E, Sata M, Ito M, Yana S. Role of monocytes in the augmentation of human natural killer activity by Interleukin-2. *Anticancer Research* 1984; **4**: 63–68.
71. Rosenstein M, Yron I, Kaufmann Y, Rosenberg SA. Lymphokine-activated killer cells: lysis of fresh syngeneic natural killer–resistant murine tumor cells by lymphocytes cultured in interleukin 2. *Cancer Res* 1984; **44**: 1946–1953.
72. Rosenberg SA, Lotze MT, Muul LM, *et al.* A progress report on the treatment of 157 patients with advanced cancer using lymphocyte-activated killer cells and interleukin-2 or high-dose interleukin-2 alone. *N Engl J Med* 1987; **316**: 889-897.
73. Stevens EJ, Jacknin L, Robbins PF, *et al.* Generation of tumor-specific CTL from melanoma patients by using peripheral blood stimulated with allogeneic melanoma tumor cell lines. Fine specificity MART-1 melanoma antigen recognition. *J Immunol* 1955; **154**: 762–771.
74. Kawakami Y, Eliyahu S, Jennings C, *et al*. Recognition of multiple epitopes in the human melanoma antigen gp100 by tumor-infiltrating T lymphocytees associated with *in vivo* tumor regression. *J Immunol* 1995; **154**: 3961–3968.
75. Negrier MS, Pourreau CN, Palmer PA, *et al*. Phase I trial of recombinant Interleukin-2 followed by recombinant Tumor Necrosis Factor in patients with metastatic cancer. *J Immunother* 1992; **11**: 93–102.
76. Wersall JP, Masucci G, Hjelm AL, *et al*. Low dose cyclophosphamide, alpha-interferon and continuous infusions of interleukin-2 in advanced renal cell carcinoma. *Med Oncol Tumor Pharmacother* 1993; **10**: 103–111.
77. Law TM, Motzer RJ, Mazudmar M, *et al*. Phase III randomized trial of Interleukin 2 with or without lymphokine-activated killer cells in the treatment of patients with advanced renal cell carcinoma. *Cancer* 1995; **76**: 824–832.
78. Bezooijen van RL, Goey H, Stoter G, Hermans J, Fleuren GJ. Prognostic markers for survival in patients with metastatic renal cell carcinoma treated with interleukin-2. *Cancer Immunol Immunother* 1996; **43**: 293–298.
79. Ting CC, Wang J, Yang Y. Interleukin-2 and interleukin-7 augment the cytotoxic activity and expand the antitumor killing spectrum of α CD3-induced activated killer cells: potential use in the immunotherapy of non-immunogenic tumors. *Cancer Immunol Immunother* 1996; **43**: 283–292.
80. Teichmann JV, Ludwig WD, Thiel E. Cytotoxicity of Interleukin 2 induced lymphokine activated killer (LAK) cells against human leukemia and augmentation of killing by interferons and tumor necrosis factor. *Leukemia Res* 1992; **16**: 287–298.
81. Mule JJ, Shu S, Schwarz SL, Rosenberg SA. Adoptive immunotherapy of established pulmonary metastases with LAK cells and recombinant Interleukin-2. *Science* 1984; **225**: 1487–1489.
82. Lotzova E, Herberman RB. Reassessment of LAK phenomenology; A review. *Nat Immun Cell Growth Regul* 1987; **6**: 109–115.
83. Lotzova E. Cytotoxicity and clinical application of activated NK cells. *Med Oncol Tumor Pharmacother* 1989; **6**: 93–98.
84. Gutman M, Laufer R, Eisenthal A, *et al*. Increased microvascular permeability induced by prolonged interleukin-2 administration is attenuated by the oxygen-free radical scavenger dimethylthiourea. *Cancer Immunol Immunother* 1996; **43**: 240–244.
85. Zeimet AG, Natoli C, Herold M, *et al*. Circulating immunostimulatory protein 90K and soluble interleukin-2 receptor in human ovarian cancer. *Int J Cancer* 1996; **68**: 34–38.
86. Gansbacher B, Zier K, Daniels B, *et al*. Interleukin 2 gene transfer into tumor cells abrogates tumorigenicity and induces protective immunity. *J Exp Med* 1990; **172**: 1217–1224.
87. Russell SJ, Eccles S, Flemming CL, *et al*. Decreased tumorigenicity of a transplantable rat sarcoma following transfer and expression of an IL-2 DNA. *Int J Cancer* 1991; **47**: 244–251.
88. Cavallo F, Giovarelli M, Gulino A, *et al*. Role of neutrophils and $CD4^{+}$ T lymphocytes in the primary and memory response to nonimmunogenic murine mammary adenocarcinoma made immunogenic by IL-2 gene. *J Immunol* 1992; **149**: 3626–3635.
89. Karp SE, Farber A, Salo JC, *et al*. Cytokine secretion by genetically modified nonimmunogenic murine fibrosarcoma. Tumor inhibition by IL-2 but not Tumor Necrosis Factor. *J Immunol* 1993; **150**: 896–901.
90. Belldegrun A, Tso CL, Sakata T, *et al*. Human renal carcinoma line transfected with Interleukin-2 and/or interferon α gene(s): implications for live cancer vaccines. *JNCI* 1993; **85**: 207–216.
91. Kikuchi Y, Imaizumi E, Kataoka Y, *et al*. Effects of granulocyte-colony-stimulating factor and Interleukin-2 on ascites formation and the survival time of nude mice bearing human ovarian cancer cells. *Cancer Immunol Immunother* 1996; **43**: 257–261.
92. Tepper RI, Pattengale PK, Leder P. Murine interleukin-4 displays potent anti-tumor activity *in vivo*. *Cell* 1989; **57**: 503–512.
93. Golumbek PT, Lazenby AJ, Levitsky HI, *et al*. Treatment of established renal cancer by tumor cells engineered to secrete Interleukin-4. *Science* 1991; **254**: 713–716.
94. Elsasser-Belle U, von Kleist S. Cytokines as therapeutic and diagnostic agents. *Tumor Biol* 1993; **14**: 69–94.
95. Mule JJ, McIntosh JK, Jablons DM, Rosenberg SA. Antitumor activity of recombinant interleukin 6 in mice. *J Exp Med* 1990; **171**: 629–636.
96. Chen L, Mory Y, Zilberstein A, Revel M. Growth inhibition of human breast carcinoma and leukemia/lymphoma cell lines by recombinant interferon-β2. *Proc Natl Acad Sci USA* 1988; **85**: 8037–8041.
97. Fujita J, Tsuiinaka T, Yano M, *et al*. Anti-interleukin-6 receptor antibody prevents muscle atrophy in colon-26 adenocarcinoma-bearing mice with modulation fo lysosomal and ATP-Ubiquitin-dependent proteolytic pathways. *Int J Cancer* 1996; **68**: 637–643.
98. Porgador A, Tzehoval E, Katz A, *et al*. Interleukin 6 gene transfection into Lewis lung Carcinoma tumor cells suppresses the malignant phenotype and confers immunotherapeutic competence against parental metastatic cells. *Cancer Res* 1992; **52**: 3679–3686.
99. Mullen CA, Coale MM, Levy AT, *et al*. Fibrosarcoma cells transduced with the IL-6 gene exhibit reduced tumorigenicity, increased immunogenicity, and decreased metastatic potential. *Cancer Res* 1992; **52**: 6020–6024.
100. Okamoto M, Oyasu R. Effect of transfected interleukin-6 in non-tumorigenic and tumorigenic rat urothelial cell lines. *Int J Cancer* 1996; **68**: 616–621.
101. Hock BH, Dorsch M, Diamantstein T, Blankenstein T. Interleukin 7 induces CD4 + T cell dependent tumor rejection. *J Exp Med* 1991; **174**: 1291–1298.
102. Brunda MJ, Luistro L, Rumennik L, *et al*. Interleukin-12: Murine models of a potent antitumor agent. In: Lotze MT, Trinchier G, Gately M, Wolf S eds. *Interleukin 12: cellular and molecular immunology of an important regulatory cytokine*. New York: Ann New York Acad Sci, 1996; **795**: 266–274.
103. Tahara H, Zitygel L, Storkus WJ, Robbins PD, Lotze MT. Murine models of cancer cytokine gene therapy using Interleukin-12. In: Lotze MT, Trinchier G, Gately M, Wolf S. eds. *Interleukin 12: cellular and molecular immunology of an important regulatory cytokine*. New York: Ann New York Acad Sci, 1996; **795**: 275–283.
104. Zitvogel L, Couderc B, Mayordomo JI, Storkus WJ. IL-12 engineered dendritic cells serve as effective tumor vaccine adjuvants *in vivo*. In: Lotze MT, Trinchier G, Gately M, Wolf S eds. *Interleukin 12: cellular and molecular immunology of an important regulatory cytokine*. New York: Ann New York Acad Sci, 1996; **795**: 284–293.
105. Fujiwara H, Clark SC, Hamaokka T. Cellular and molecular mechanisms underlying IL-12-induced tumor regression. In: Lotze MT, Trinchieri G, Gately M, Wolf S eds. *Interleukin 12. Cellular and molecular Immunology of an important regulatory cytokine*. New York: Ann New York Acad Sci, 1996; **795**: 294–309.
106. Lotze MT, Zitvogel L, Campbell R, *et al*. Cytokine gene therapy of cancer using Interleukin-12: murine and clinical trials. In: Lotze MT, Trinchieri G, Gately M, Wolf S eds. *Interleukin-12. Cellular and molecular Immunology of an important regulatory cytokine*. New York: Ann New York Acad Sci, 1996; **795**: 440–454.
107. Zitvogel L, Tahara H, Robbins PD, *et al*. Cancer immunotherapy of established tumors with interleukin-12: Effective delivery by genetically engineered fibroblasts. *J Immunol* 1995; **155**: 1393–1403.
108. Tahara H, Lotze MT. IL-12 therapy using direct injection of tumors with genetically engineered autologous fibroblasts. *Hum Gene Ther* 1995; **6**: 1067–1624.
109. Tsavaris N, Baxevanis K, Kosmidis P, Papamichael M. The prognostic significance of immune changes in patients with renal cancer melanoma treated with interferon-$\alpha_{2b}$. *Tumor Biol* 1995; **16**: 365–373.
110. Strander H. Interferons: Antineoplastic drugs? *Blut* 1977; **35**: 277–288.
111. Mellsstedt H. Biorkholm B, Johansson A, *et al*. Interferon therapy in myelomatosis. *Lancet* 1979; **I**: 245–247.
112. Merigan TC, Sikora K, Breeden JH, *et al*. Preliminary observations on the effect of human leukocyte interferon in non Hodgkin's lymphoma. *New Engl J Med* 1979; **299**: 1949–1453.
113. Gutterman J, Yap Y, Buzdar R, *et al*. Leukocyte interferon (IF) induced tumor regression in patients with breast cancer and B cell neoplasma. *Proc Am Assoc Cancer Res* 1979; **20**: 167–168.
114. Krown S, Stoopler M, Gralla R, *et al*. Phase II trial of human leukocyte interferon in non-small cell lung cancer. In: Terry WD ed. *Immunotherapy of cancer: Present status of trials in man*. New York: Raven Press, 1982.
115. Halme M, Maasilta P, Repo H, *et al*. Subcutaneously administered recombinant interferon-γ in humans: Pharmacokinetics and effects on chemiluminescence responses of alveolar macrophages, blood neutrophils and monocytes. *J Immunother* 1994; **13**: 283–291.
116. Li J, Yang Y, Inoue H, *et al*. The expression of costimulatory molecules CD80 and CD86 in human carcinoma cell lines: its regulation by interferon γ and interleukin-10. *Cancer Immunol Immunother* 1996; **43**: 213–219.
117. Watanabe Y, Kuribayashi K, Miyatake S, *et al*. Exogenous expression of mouse interferon gamma cDNA in mouse neuroblastoma C1300 cells results in reduced immunogenicity by augmented anti-tumor immunity. *Proc Natl Acad Sci USA* 1989; **86**: 9456–9460.
118. Gansbacher B, Bannerii R, Daniels B, *et al*. Retroviral vector-mediated gamma-interferon gene transfer into tumor cells generates potent and long lasting antitumor immunity. *Cancer Res* 1990; **50**: 7820–7825.
119. Esumi N, Hunt B, Itava T, Frost P. Reduced tumorigenicity of murine tumor cells secreting γ-interferon is due to non specific host responses and is unrelated to Class I major histocompatibility complex expression. *Cancer Res* 1991; **51**: 1185–1189.
120. Porgador A, Bannerii R, Watanabe Y, *et al*. Antimetastatic vaccination of tumor-bearing mice with two types of IFN-gamma gene-inserted tumor cells. *J Immunol* 1993; **150**: 1458–1470.
121. Fieren MJWA, Bemd van den GJCM, Bonta IL. Endotoxin stimulated peritoneal macrophages obtained from Continuous Ambulatory Peritoneal Dialysis patients show *in vitro* an increased capacity to release interleukin-1β during infectious peritonitis. *Eur J Clin Invest* 1990; **20**: 453–457.
122. Fieren MJWA, Bemd van den GJCM, Bonta IL, Ben-Efraim S. Peritoneal macrophages from patients on Continuous Ambulatory Peritoneal Dialysis have an increased capability to release tumour necrosis factor during peritonitis. *J Clin Lab Immunol* 1993; **34**: 1–9.
123. Spengler RN, Spengler ML, Lincoln P, *et al*. Dynamics of dibutyryl cyclic AMP and prostaglandin E2 mediated suppression of lipopolysaccharide-induced tumor necrosis alpha gene expression. *Inf Immun* 1989; **57**: 2837–2841.
124. Shalaby MR, Waage A, Aarden L, Espevik T. Endotoxin, Tumor necrosis Factor-α and Interleukin-1 induce Interleukin-6 *in vivo*. *Clin Immunol Immunopath* 1989; **53**: 488–498.
125. Schindler R, Mancilla J, Endress S, *et al*. Correlations and interactions in the production fo Interleukin-6 (IL-6), IL-1 and Tumor Necrosis Factor (TNF) in human blood mononuclear cells: IL-6 suppresses IL-1 and TNF. *Blood* 1990; **75**: 40–47.
126. Skyes JAC, Maddox IS. Prostaglandin production by experimental tumors and effects of anti-inflammatory compounds. *Nature New Biology* 1972; **237**: 59–61.
127. Furuta Y, Hall ER, Sanduka S, *et al*. Prostaglandin production by murine tumors as predictor for therapeutic response to indomethacin. *Cancer Res* 1988; **48**: 3002–3007.
128. Lonroth Chr, Moldawer LL, Gelin J, *et al*. Tumor necrosis factor-α and interleukin-1α production in cachectic tumor-bearing mice. *Int J Cancer* 1990; **46**: 889–896.
129. Hata H, Matsuzaki H, Takasuki K. Autocrine growth by two cytokines, interleukin-6 and tumor necrosis factor-α in the myeloma cell line KHM-1A. *Acta Hematol* 1990; **83**: 133–136.
130. Schoot van der CA, Jansen P, Poorter M, *et al*. Interleukin-6 and interleukin-1 production in actue leukemia with monocytoid differentiation. *Blood* 1989; **74**: 2081–2087.
131. Vincent JE, Vermeer MA, Kort WJ, Ziilstra JF. The formation of thromboxane B2, leukotriene B4, and 12-hydroxysatetraenoic acid by alveolar macrophages after activation during tumor growth in the rat. *Biochem Biophys Acta* 1990; **1042**: 255–258.
132. Parhar RS, Lala PK. Prostaglandin E2-mediated inactivation of various killer lineage cells by tumor-bearing host macrophages. *J Leuk Biol* 1988; **44**: 474–484.
133. Palleroni AV, Varesio L, Wright RB, Brunda MJ. Tumoridical alveolar macrophages and tumor infiltrating macrophage cell lines. *Int J Cancer* 1991; **49**: 296–302.
134. Moldawer LL, Lonroth C, Mizzi SB, Lunholm KG. Down-regulation of interleukin-1 production by macrophages of sarcoma-bearing mice. *J Immunol* 1987; **138**: 4270–4272.
135. Economou JS, Colquhon SD, Anderson TM, *et al*. Interleukin-1 and tumor necrosis factor production by tumor-associated mononuclear leukocytes and peripheral mononuclear leukocytes in cancer patients. *Int J Cancer* 1988; **42**: 712–714.
136. Zielinsky CC, Mueller C, Tyl E, *et al*. Imparied production of tumor necrosis factor in breast cancer. *Cancer* 1991; **68**: 1944–1948.
137. Barra BP, Rogers LR, Thomassen MJ, *et al*. Monocyte tumoricidal activity and tumor necrosis factor production in patients with malignant brain tumors. *Cancer Immunol Immunother* 1991; **33**: 314–318.
138. Ikemoto S, Kishimoto T, Nishio S, *et al*. Correlation of tumor necrosis factor and prostaglandin E2 production of monocytes in bladder cancer patients. *Cancer* 1989; **64**: 2076–2080.
139. Cortesina G, De Stefani A, Giovarelli M, *et al*. Treatment of recurrent squamous cell carcinoma of the head and neck with low doses of interleukin-2 injected perilymphatically. *Cancer* 1988; **62**: 2482–2485.
140. Gastl G, Finstad CL, Guarini A, *et al*. Retroviral vector-mediated lymphocine gene transfer into human renal cancer cells. *Cancer Res* 1992; **52**: 6226–6236.
141. Cignetti A, Guarini A, Carbone A, *et al*. Transduction of the IL-2 gene into human acute leukemia cells induces tumor rejection without modifying cell proliferation and IL-2 receptor expression. *JNCI* 1994; **86**: 785–790.
142. Forni G, Foa R. The role of cytokines in tumor rejection. In: Dagleish AG, Browning MJ eds. *Tumor Immunology. Immunotherapy and cancer vaccines*. Cambridge: Cambridge University Press, 1996; 199–218.
143. Herrmann F. Cytokines in cancer therapy. *J Cancer Res Clin Oncol* 1989; **115**: 101–104.
144. Di Pierro F, Cavallo F, Pericle F, *et al*. Strategies for cytokine utilisation in tumor therapy. *Med Oncol Tumor Pharmacother* 1993; **10**: 53–59.

ACKNOWLEDGEMENTS. The work of the author and his coassociates, reported in this review, was supported by research grants from: the Dutch Cancer Foundation (Konigin Wilhelmina Fonds); Erasmus University Foundation (Stichting Universiteit Fonds Rotterdam); Supporters of the Joint Israel–Dutch Medical Research (under the auspices of the Israeli Cancer Association, Tel-Aviv, Israel; Emil Starkenstein Foundation; Rotterdam; and an endowment made by Meir and Rebeca Heinik in memory of their son Joseph Heinrichson.

**Received 9 April 1997; accepted 10 April 1997**
